# Long-term effects of altitude on changes in intestinal flora in a young and middle-aged population: a longitudinal analysis before and after 6 months of entry to high altitude

**DOI:** 10.3389/fmicb.2025.1699737

**Published:** 2026-01-02

**Authors:** Hongjiao Kan, Yingna Yuan, Kaijie Zhao, Xiuting Sun, Junlian Li, Farong Huo, Xiaoyan Zhang

**Affiliations:** Department of Neurology, The 940th Hospital of Chinese People's Liberation Army Joint Logistic Support Force, Lanzhou, China

**Keywords:** high altitude, young and middle-aged, intestinal flora, metabolites, 16S rRNA

## Abstract

**Introduction:**

The low oxygen environment of high altitude predisposes to imbalances in gut flora and changes in metabolites that can affect metabolic health. The relationship between gut flora and the health of sedentary populations at high altitude has been extensively studied. However, the dynamic changes of gut flora and its metabolomics after entering high altitude and the long-term effects on the health of young and middle-aged people have not been studied enough. This study aims to investigate the patterns of gut microbiota and metabolomics changes in middle-aged and young adults during the week preceding ascent to 4,500 m above sea level and within the 6 months following high-altitude exposure.

**Methods:**

A total of 70 healthy young and middle-aged people were collected from November 2023 to June 2024 from an altitude of about 1,600 to 4,500 m, and their stool samples were collected. 16S rRNA sequencing and liquid-liquid-mass spectrometry (LC-MS/MS) were used to detect the changes in the composition of the gut microorganisms and the differential metabolites of the intestinal tract in the young and middle-aged people before and after they entered the altitude, and the changes in the abundance and composition of the gut flora and their metabolomics were analyzed. Abundance and composition and the changes in their metabolomics.

**Results:**

The results showed that the largest differences in the abundance of intestinal microorganisms at the phylum and genus levels were Firmicutes and *Blautia* in High-altitude group (HG) compared with Low-altitude group (PG), and the alpha diversity (α-diversity) and abundance of intestinal microorganisms in HG were lower than those in PG. The differences in the flora at the phylum level were significantly higher in the Actinobacteriota (Actinobacteria) relative abundance of HG compared with that of PG, and the relative abundance of PG was higher in the Actinobacteria (Actinobacteria) phylum than that of PG. Actinobacteriota (phylum) relative abundance was significantly higher in HG compared to PG (*p* < 0.001). At the genus level, the relative abundance of *Blautia, Bifidobacterium, Fusicatenibacter*, and *Anaerestipes* was significantly higher in HG compared to PG (*p* < 0.05 or *p* < 0.001). The compound classes with the largest ratio of HG to PG were Lipids and lipid-like molecules (25.15%), Lipids and lipid-like molecules (25.15%), and Lipids and lipid-like molecules (25.15%). There was a correlation between differential metabolites in HG and PG samples. Volcano plots showed 456 up-regulated and 425 down-regulated metabolites were identified for HG compared to PG. Six major classes of metabolic pathways were detected for KEGG.

**Discussion:**

In conclusion, significant changes in the diversity, abundance, composition and differential metabolites of the intestinal gut flora occurred before and after entering high altitude in young and middle-aged populations, which may be important for the adaptation of young and middle-aged populations in the plateau environment.

## 1 Introduction

The human microbiota contains a huge number of microorganisms, among which bacteria are the most abundant (Qiu et al., 2022). Intestinal flora play a variety of important roles in human physiological activities, including participation in food digestion and nutrient absorption, synthesis of vitamins and other beneficial substances, regulation of the immune system, and maintenance of the intestinal barrier function (Silva et al., 2020). The balance of intestinal flora is essential for maintaining human health, and once the flora is imbalanced, it may lead to various diseases, such as infection, immunity, obesity, diabetes, cardiovascular diseases, and intestinal inflammatory diseases (Guo et al., 2025). Studies have found that the health of intestinal flora is closely related to a variety of factors, including the following: diet (David et al., 2014), antibiotic use (Jernberg et al., 2010), age (Ling et al., 2022), environmental factors (Yatsunenko et al., 2012), lifestyle (Clauss et al., 2021), psychological stress (Foster and Mcvey Neufeld, 2013), as well as diseases and infections (Ding et al., 2019). These factors interact with each other to influence the diversity and function of the gut microbiota, which in turn has a profound effect on the immune, metabolic, and neurological functions of the host.

At high altitudes, unique environmental conditions such as low barometric pressure, low partial oxygen pressure, low humidity, low temperature, and high ultraviolet radiation have a significant impact on the lives and health of people living in highland areas. In this environment, the composition of the intestinal flora changes, and these changes are closely related to the health and adaptation of the host (Su et al., 2024b). The low-pressure and low-oxygen conditions in high-altitude environments alter the oxygen content in the gut, which in turn affects the growth and reproduction of aerobic and parthenogenetic anaerobic bacteria. At the same time, this environmental change may also have an indirect effect on the intestinal flora by affecting intestinal blood circulation and intestinal mucosal function (Liu et al., 2024). During rapid ascent to high altitude, the integrity of the gastric mucosal barrier function is impaired by low pressure and oxygen at high altitude, which disrupts the balance of intestinal flora and leads to metabolic disorders (Suzuki et al., 2019). However, a review of previous relevant studies found that short-term exposure to hypoxia at high altitude significantly altered the intestinal flora composition (Su et al., 2024b). One study assessed the changes in the intestinal flora of mice acutely exposed to simulated high-altitude hypoxia conditions, and found that the diversity and composition of the intestinal flora of mice in the hypoxia group changed significantly compared with that of the normoxic group, which may be related to the adaptation of the host to the high-altitude environment (Wang et al., 2022). It was noted that gut flora can indirectly regulate the host's blood pressure and energy intake efficiency, and thus may contribute to the maintenance of normal energy production and/or optimization of nutrient absorption in high-altitude hypoxic environments (Mazel, 2019). The structure of the gut flora of high-altitude residents or those who have been exposed to high-altitude environments for a long period differs from that of low altitude (Han et al., 2024). The effects of long-term high-altitude exposure on the intestinal flora show adaptive changes that may be related to oxygen utilization, immune regulation, metabolic homeostasis and other factors. The composition and function of intestinal flora may be significantly altered by long-term exposure to low oxygen. Previous studies of long-term high-altitude exposure have been dominated by studies of changes in animal intestinal flora, and most of these studies have been compared with long-term high-altitude residents.

Long-term exposure to high altitude helps the host adapt to extreme environments such as low oxygen and low temperature by adjusting metabolic pathways and regulating key metabolites. A longitudinal multi-omics study (Han et al., 2024) found that the intestinal flora of the Han Chinese population gradually converged to the characteristics of the Tibetan population after entering high altitude, while metabolites such as lactate and taurine were significantly elevated in plasma, suggesting that flora-driven metabolic reprogramming may enhance hypoxic adaptation by regulating energy metabolic pathways. Prolonged plateau exposure activated energy metabolic pathways (e.g., vitamin synthesis and lipid metabolism) with concomitant increases in the abundance of butyric acid-producing bacteria (e.g., *Trichoderma* sp. Lachnospiraceae), and butyric acid mitigated intestinal damage by inhibiting hypoxia-inducible factor (HIF-1α) expression (Zhao et al., 2024). Increased abundance of uric acid-degrading bacteria (e.g., Lachnospiraceae) in the gut of long-term plateau populations leads to lower gut uric acid levels, which may facilitate hypoxic adaptation by reducing oxidative stress (Su et al., 2024a).

Young and middle-aged people, as the main labor force and activity group in society, are more likely to enter high-altitude areas for work, travel and other reasons. Their health status in high-altitude environments is not only related to their quality of life and work efficiency, but also of great significance to the development of society. However, most of the current studies have the problems of small sample size and short study period, and there are insufficient studies on the dynamic changes and long-term effects of intestinal flora and its metabolomics in young and middle-aged people after entering high altitude, and there is a lack of in-depth discussion on the causal relationship between changes in intestinal flora and its metabolomics and clinical symptoms and disease development. Longitudinal studies of the gut microbiota in specific populations are essential to reconstruct the changes in the gut and its metabolomics during high-altitude exposure. Based on this, the present study aims to investigate the dynamics of the gut flora and its metabolic patterns after entering high altitude in young and middle-aged populations, including the changes in flora structure, diversity, and function, as well as the relationship between these changes and human physiological functions, which can help to identify the potential core microbiota that may contribute to the adaptation of the host to the hypoxic extremes of the environment. This study is important for revealing the adaptation mechanism of gut flora in young and middle-aged people in high-altitude environments, and provides a basis for the prevention and treatment of high-altitude related diseases and the development of targeted health protection measures. It may provide new ideas for the development of microecological agents based on the regulation of intestinal flora for the prevention and treatment of high altitude diseases.

## 2 Methods

### 2.1 Survey participants

Seventy healthy young and middle-aged people who entered the area of 4,500 m above sea level from 1,500 m above sea level from November 2023 to June 2024 were randomly selected for this study. Inclusion criteria were as follows: age ≥18 years, and all participants were in good health. Exclusion criteria were as follows: those who were taking antibiotics, microbial modifiers such as probiotics and prebiotics, immunosuppressants, and special medications within the last 2 months. Demographic information of the participants was collected. There were 70 participants, mean age 35.57 ± 7.66 years, mean height 1.69 ± 0.08 m, weight 65.25 ± 11.31 kg; BMI: 22.81 ± 2.51 kg/m^2^. Participants were 33 females and 37 males. Among them, one female participant was Tibetan, one male participant was of the She ethnic group, and the rest were all Han. All the subjects had similar living environments and dietary habits. All participants read and signed the informed consent form. The Ethics Committee of the 940th Hospital of the Chinese People's Liberation Army (Lanzhou, China) evaluated and approved the study (2023KYLL308).

### 2.2 Research methods

#### 2.2.1 Fecal samples acquisition

General data collection and fecal sample collection were collected from all participants 1 week before and 6 months after entering the plateau, respectively. Throughout the sampling process, participants were fed a uniform diet, maintained a similar work schedule, and had a similar level of activity, which largely minimized the effects of lifestyle. Dietary details for the study population: maintain daily energy intake around 3,500 kcal. Breakfast: noodles, eggs, and greens (shredded radish or potato), and milk (approx. 580 kcal); Lunch: steamed buns, meat, and vegetables (celery, broccoli, and Chinese cabbage), and one apple (approx. 1,430 kcal); Dinner: steamed buns, meat, and vegetables (stir-fried mustard greens, eggplant, and tomatoes with eggs), and one orange or tangerine (approx. 1,440 kcal). Stool sample collection method: participants were instructed to empty their bladders prior to stool sample collection and to wear disposable gloves during sampling to avoid direct contact with the sample. Stool samples were collected using a special DNA-free, sterile stool sample collection tube. After the first bowel movement in the early morning, the participants were asked to collect a stool sample from the center of the stool, which was not in contact with water, urine or air, using a sterile sampling spoon in the stool sample collection tube, and the sample was taken from the center of the stool, which was not in contact with water, urine or air, and the total amount of the sample was about 3 g. The sample was then transferred to a refrigerator and stored in the refrigerator at −80 °C for 2 h until analysis.

#### 2.2.2 DNA extraction and PCR amplification

The samples were transported at low temperatures, and in the DNA extraction process, the genomic DNA of the fecal samples was extracted by the pentadecyltrimethylammonium bromide (CTAB) method to check its purity and concentration. The extraction process included sample pretreatment, lysis, impurity removal, binding, washing and elution. Specifically, about 200 mg of fecal sample was first placed in a 2 mL centrifuge tube, added with the appropriate lysis solution and vortexed for mixing, and then processed in a water bath, and then centrifuged to remove impurities, and then the DNA adsorbent column and eluent were used to complete the binding, washing and elution of DNA, so that high-purity and stable DNA was extracted, which is suitable for subsequent experiments such as enzyme digestion, PCR, library construction, and other experiments.

For PCR amplification, the V3–V4 region of the 16S rRNA gene is amplified, and the company uses specific primers and high-fidelity heat-stable DNA polymerase for amplification. The PCR reaction system contains buffer, dNTPs, primers and template DNA, etc. The reaction process is carried out in a thermal cycler, including initial denaturation, denaturation with multiple cycles, annealing and extension steps, and final extension. The amplification products were verified and purified by 2% agarose gel electrophoresis, and the qualified samples were used for subsequent library construction. Sequencing was performed on the Illumina NovaSeq 6000 platform. Raw data obtained from Illumina's MiSeq platform were excised using the QIIME2 cutadapt plug-in. Amplicon sequence variants (ASVs) were obtained after QIIME2 Dada2 quality control and denoising, and the ASV feature sequences were compared with the template sequences in the corresponding databases to obtain the corresponding taxonomic information for each ASV. Primers were designed and synthesized by Novozymes.

#### 2.2.3 Metabolomics detection and analysis

Stool sample preparation for ultra-high performance liquid chromatography. Ultra-high performance liquid chromatography (UHPLC) was coupled with high-resolution mass spectrometry, i.e., liquid-liquid-mass spectrometry (LC-MS/MS) technology, and this project was based on liquid-liquid-mass spectrometry (LC-MS) technology to conduct non-targeted metabolomics research, and the experimental procedure mainly included metabolite extraction of the samples, LC-MS/MS detection and data analysis etc. The samples were collected and stored at −80 °C. Stool samples were collected and stored at −80 °C. After thawing, about 0.1–0.2 g of the sample was weighed into a centrifuge tube, and pre-cooled extraction solvent (e.g., methanol or acetonitrile) was added to the sample and mixed by vortexing and shaking. This was followed by sonication in an ice-water bath to release metabolites, and then centrifugation at high speed at 4 °C to precipitate impurities. The supernatant was taken and dried under a stream of nitrogen, then re-solubilized with a re-solvent and filtered, and finally the sample was injected into an autosampling vial for UHPLC-MS analysis.

### 2.3 Statistical methods

Statistical analyses were performed using SPSS 25.0 software and the QIIME 2 analysis platform, and graphs were plotted by the R package; measurements that conformed to normal distribution were expressed as ($\tilde{x}\pm$ s). The α-diversity indexes of the intestinal flora (including Chao1 index, Ace index, Shannon index, and Simpson index) were further analyzed in the QIIME 2 platform, in which the higher Chao1 index and Ace index indicated the higher total number of species of intestinal flora, and the higher Shannon index and the lower Simpson index indicated the richer diversity of intestinal flora; and The beta diversity of the intestinal flora was analyzed based on the Bray-Curtis distance. Alpha diversity was analyzed by Wilcoxon test; beta diversity was analyzed by ANOSIM comparative analysis; and the differences among bacteria were analyzed by the Kruskal–Wallis rank-sum test. *p* < 0.05 was taken as the difference was statistically significant. The relative abundance of various genera was statistically analyzed by clustering for the features obtained from sequencing and the data were clustered to microbial genera, and their diversity, differential bacterial groups, inter-species correlation, and correlation between differential bacterial groups and diseases were also analyzed.

The identified metabolites were annotated using the KEGG database, the HMDB database and the LIPIDMaps database. In the multivariate statistical analysis section, the data were transformed using the metabolomics data processing software metaX and subjected to principal component analysis (PCA) and partial least squares-discriminant analysis (PLS-DA) to derive the VIP value for each metabolite. In the univariate analysis section, statistical significance (*p*-value) of each metabolite between the two groups was calculated based on the *t*-test, and the fold change of metabolites between the two groups was calculated, i.e., FC value. The default criteria for differential metabolite screening were VIP > 1, *p*-value <0.05 and FC ≥ 2 or FC ≤ 0.5. Volcano plots, plotted with the R package ggplot2, allowed for the combination of the metabolite's VIP value, log_2_ (Fold Change), and –log_10_ (*p*-value) parameters to screen metabolites of interest. Clustering heatmaps, plotted with the R package Pheatmap, were used to normalize the metabolite data using *z*-score. Correlation analysis (Pearson correlation coefficient) between different metabolites was performed using the R language cor(), statistical significance was achieved by cor.mtest() in R. A *p*-value of <0.05 was considered to be statistically significant, and correlation maps were plotted using the corrplot package in R. Bubble plots were performed with the R package ggplot2, and the KEGG database was used to study the function and metabolic pathways of metabolites, which were considered to be enriched when x/n>y/n, and significantly enriched when the *p*-value of the metabolic pathway was <0.05.

## 3 Results

### 3.1 Characteristics of microbiological changes at different stages before and after high altitude exposure in young and middle-aged populations

#### 3.1.1 Changes in the composition and structure of intestinal flora between HG and PG

**Figure 1:**
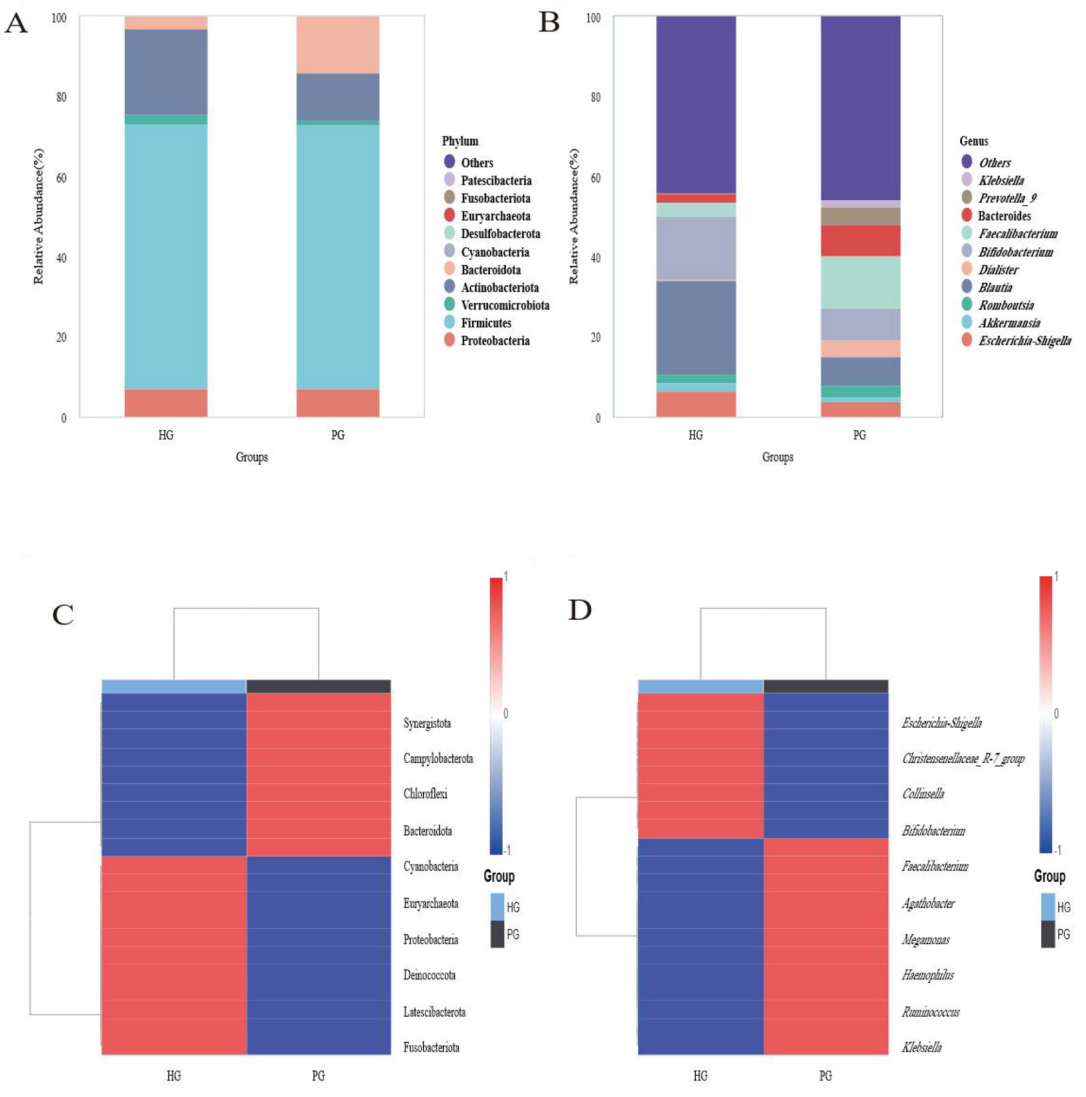
Comparison of gut flora composition before and after high altitude exposure. **(A)** Comparison of intestinal flora at the portal level. **(B)** Comparison of intestinal flora at the genus level. **(C)** Heat map of intestinal flora at the portal level. **(D)** Heat map of intestinal flora at the genus level.

At the phylum level, we analyzed the relative abundance of the top 10 bacterial phyla in the gut microbiota. As shown in Figure 1A, the following phyla exhibited abundance differences between the HG and PG groups: Firmicutes (66.32% vs. 65.86%), Verrucomicrobiota (2.15% vs. 1.36%), Actinobacteriota (21.51% vs. 11.6%), Proteobacteria (7.08% vs. 7.05%), Bacteroidota (2.84% vs. 13.86%), Cyanobacteria (0.07% vs. 0.01%), and Euryarchaeota (0.02%).

At the genus level, the top 10 bacterial genera with high relative abundance in the gut microbiota were analyzed. As shown in Figure 1B, genera exhibiting abundance differences between the HG and PG groups included: *Blautia* (23.55% vs. 6.96%), *Bifidobacterium* (15.59% vs. 7.95%), *Escherichia-Shigella* (6.38% vs. 3.74%), *Akkermansia* (2.15% vs. 1.36%), *Faecalibacterium* (3.62% vs. 13.09%), *Bacteroides* (2.25% vs. 7.57%), *Romboutsia* (2.18% vs. 2.99%), *Dialister* (0.17% vs. 4.26%), *Prevotella_9* (0.12% vs. 4.52%), and *Klebsiella* (0.23% vs. 1.78%).

At the phylum level, the correlation heatmap analyzing the top 10 bacterial phyla with high relative abundance in the HG and PG groups is shown in Figure 1C. HG exhibited a significant negative correlation with Synergistota, Campylobacterota, and Chloroflexi (*r* = −0.71, *p* < 0.001), while showing a significant positive correlation with Cyanobacteria, Euryarchaeota, Deinococcota, Latescibacterota, and Fusobacteriota (*r* = 0.71, *p* < 0.001). Conversely, PG exhibited opposite correlations with HG. At the genus level, Figure 1D shows a correlation heatmap analyzing the top 10 bacterial genera with high relative abundance in both HG and PG groups. HG showed a significant positive correlation with *Christensenellaceae-R-7* (*r* = 0.71, *p* = 0.014) and a significant negative correlation with *Faecalibacterium, Agathobacter, Megamonas, Haemophilus, Ruminococcus*, and *Klebsiella* (*r* = 0.71, *p* = 0.036, 0.016, <0.001, <0.001, 0.018, 0.002), while PG showed a significant negative correlation with *Christensenellaceae-R-7* (r = −0.71, *p* = 0.002) and a significant positive correlation with *Agathobacter, Megamonas, Haemophilus, Ruminococcus*, and *Klebsiella* (*r* = 0.71, *p* = 0.044, 0.008, 0.009, 0.022, 0.018).

#### 3.1.2 Changes in gut flora diversity between HG and PG

**Figure 2:**
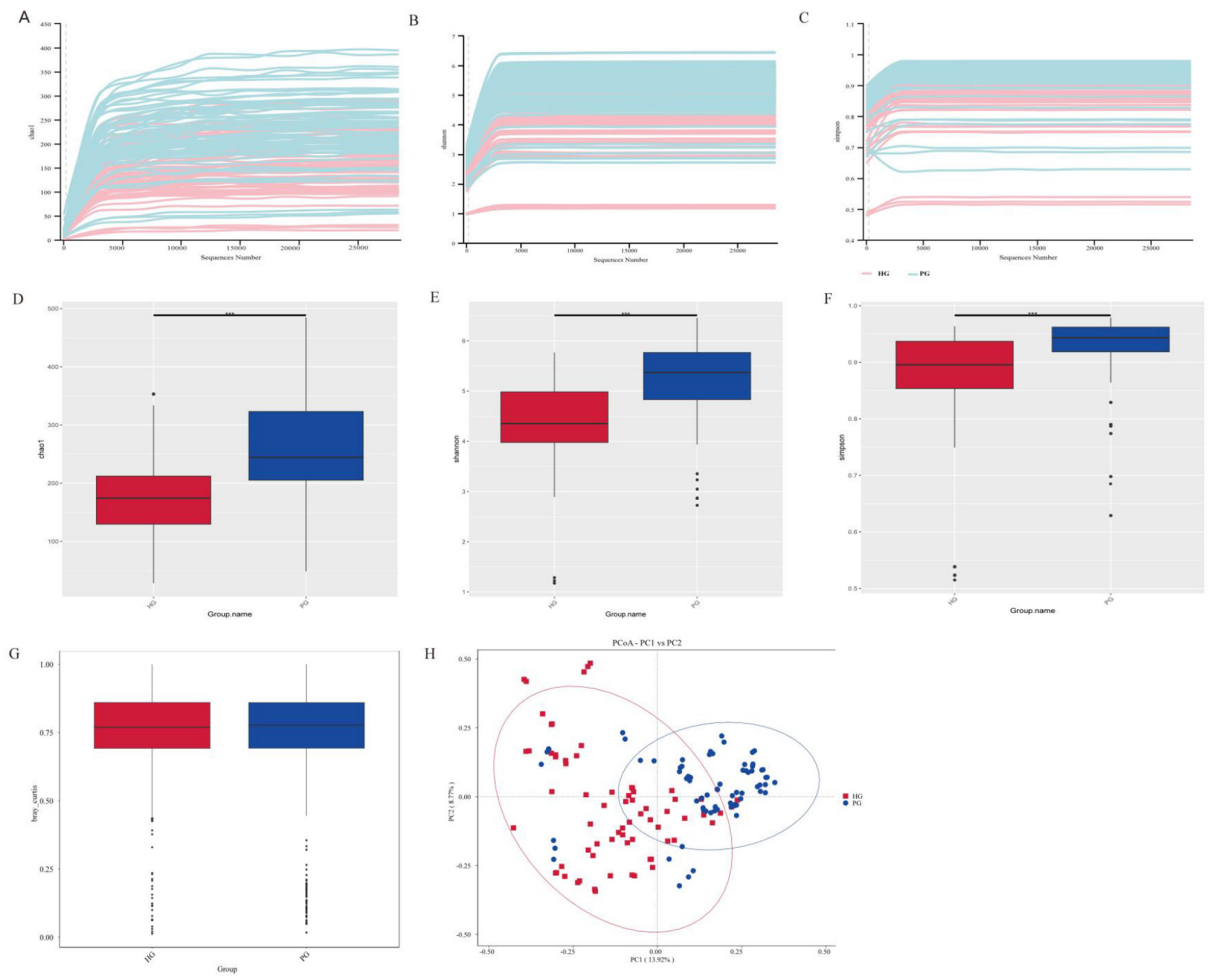
Comparison of intestinal flora diversity before and after high altitude exposure in the young and middle-aged population. **(A)** α-diversity dilution curve-Chao1 index. **(B)** α-diversity dilution curve-Shannon index. **(C)** α-diversity dilution curve-Simpson index. **(D)** α-diversity of HG vs. PG-Chao1 index. **(E)** α-diversity of HG vs. PG-Shannon index. **(F)** α-diversity of HG vs. PG-Simpson index. **(G)** β-diversity of HG vs. PG. **(H)** PCOA analysis based on the Bray-Curtis algorithm. ***p < 0.05.

Plotting the dilution curves of gut microbial α-diversity (Chao1 index, Shannon index, Simpson index) between HG and PG (Figures 2A–C) showed that the dilution curves of the two groups tended to flatten out and approach the plateau period when the sequencing depth was up to 5,000, which determined that the depth of sequencing of gut microbial α-diversity of the two groups in the present study was sufficient.

Comparison of α-diversity between the two groups of HG and PG, Figure 2D shows the Chao1 index, Figure 2E shows the Shannon index, and Figure 2F shows the Simpson index, and it was found that there was a significant difference between the two groups of Chao1, Shannon, and Simpson indexes (*p* < 0.001), and that HG had a lower diversity and abundance of gut microbes than PG. The present study was based on the Bray-Curtis algorithm to assess the β-diversity between the two groups of HG and PG. Figure 2G shows the β-diversity between the two groups, and Figure 2H shows the PCoA plot between the two groups. It was found that there was no significant difference in β-diversity between HG and PG, and PCoA analysis showed that HG gut microbial clusters were more distributed compared to PG.

#### 3.1.3 Analysis of differential flora between HG and PG

**Figure 3:**
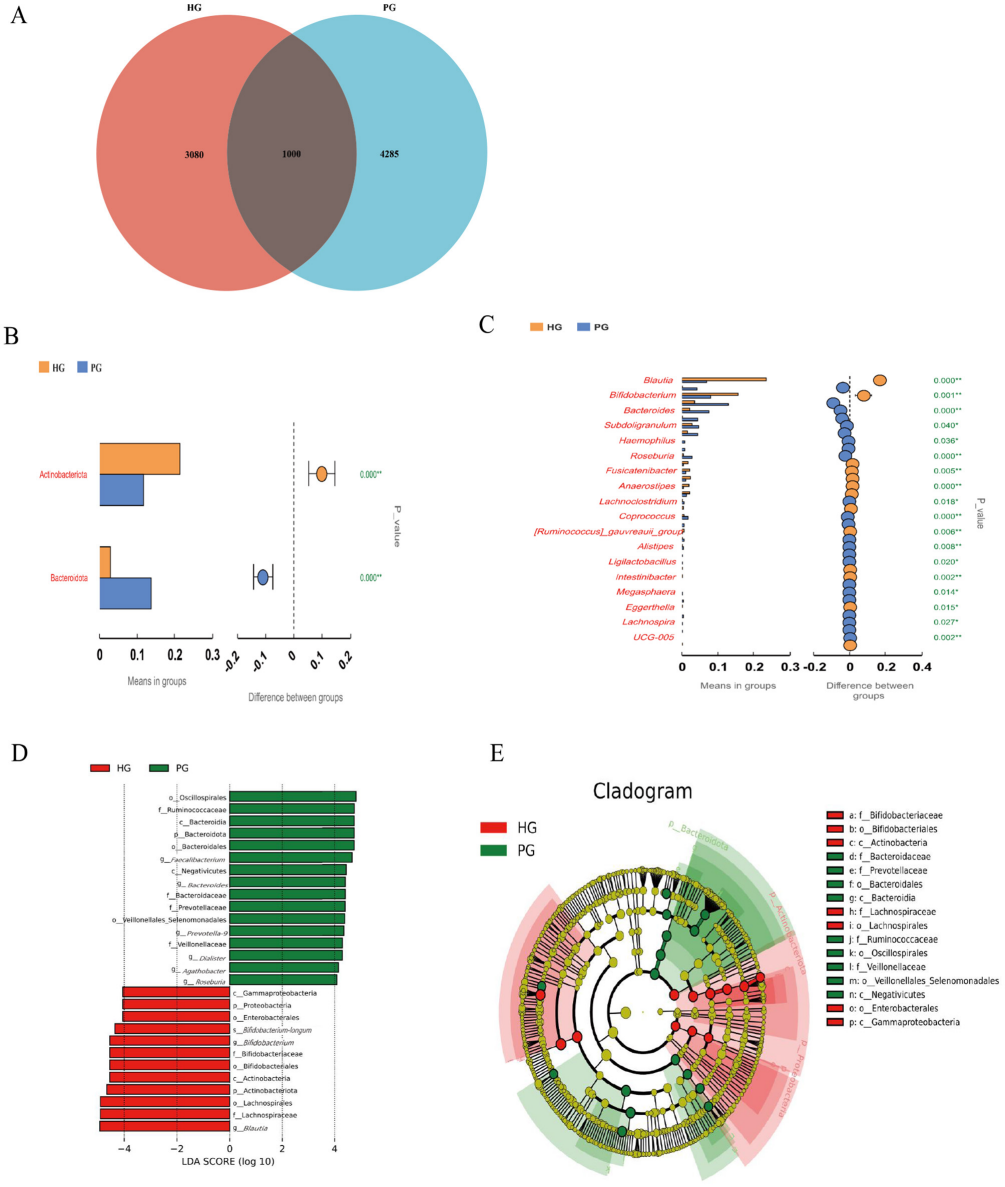
Analysis of differential flora before and after high altitude exposure in young and middle-aged populations. **(A)** Venn diagram of HG vs PG. **(B, C)** Differences in gut microbiota between HG and PG were detected by a Kruskal–Wallis rank-sum test for gut flora at the phylum **(B)**, genus **(C)** levels. **(D)** Plot of LEfSe analysis between HG and PG (LDA value >4, *p* < 0.05). **(E)** Evolutionary branching plot between HG and PG.

Venn diagrams were used to show the number of shared and exclusive ASVs between the two different groups of HG and PG (Figure 3A). The results showed that there were 1,000 ASVs shared between HG and PG. In total of 3,080 ASVs were unique to HG, and 4,285 ASVs were unique to PG.

To investigate the differential abundance of gut microbial taxa between HG and PG, we used the Kruskal-Wallis rank-sum test method to compare the differences in gut flora abundance between the two groups at the phylum level and genus level. Significant differences were found in two phylum and 18 genera (Figures 3B, C). At the phylum level, the relative abundance of Actinobacteriota (Actinobacteria) was significantly higher in HG compared to PG (*p* < 0.001), whereas the relative abundance of Bacteroidota was significantly lower in HG (*p* < 0.001). At the genus level, the relative abundance of Blautia, Bifidobacterium, Fusicatenibacter, and Anaerestipes was significantly higher (*p* < 0.05 or <0.001) in HG compared to PG. To analyze the statistical differences in microbial communities between HG and PG, we also performed LEfSe analyses (Figures 3D, E) and plotted the branching diagrams of taxa with LDA values of 4.0 (Figure 3D). From the LEfSe analysis of evolutionary branching maps, this analysis showed that the taxa with the highest differential abundance were enriched in gut microbiota, compared to PGs, HGs of family Bifidobacteriaceae, family Lachnospiraceae, order Bifidobacteriales, order Lachnospirales, order Enterobacterales, class Actinobacteria, and class Gammaproteobacteria had higher abundance. However, HG's family Bacteroidaceae, family Prevotellaceae, family Ruminococcaceae, family Veillonellaceae, order Bacteroidales, order Veillonellales- Selenomonadales, order Oscillospirales, class Bacteroidia, class Negativicutes were significantly less abundant.

#### 3.1.4 Analysis of differential metabolites between HG and PG gut microbiota

**Figure 4:**
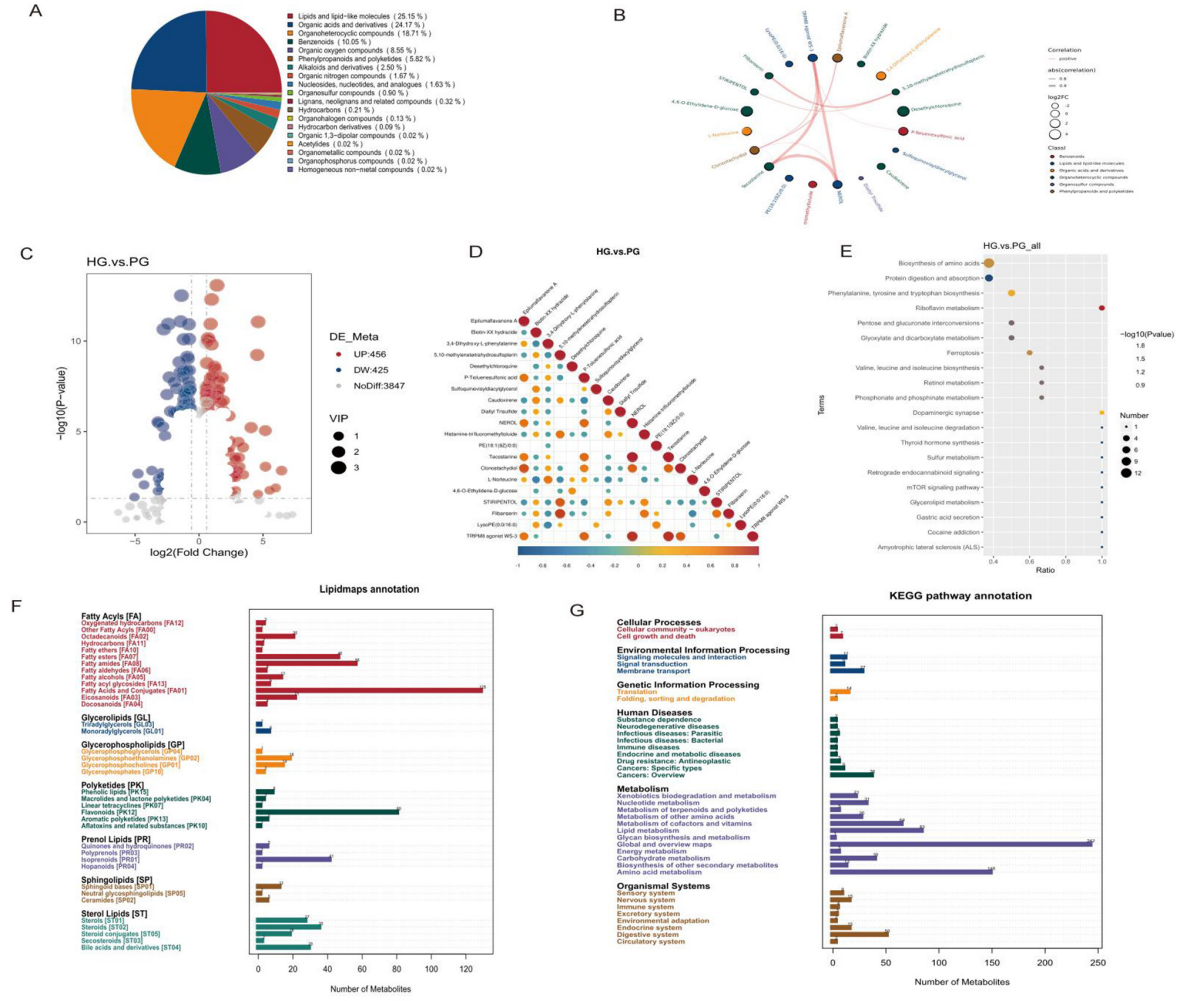
Differential metabolite analysis before and after high altitude exposure in young and middle-aged populations. **(A)** Dendrogram analysis based on gut microbiota metabolites: Human Metabolome Database (HMDB) compound classification of metabolites with significant differences between HG and PG groups at the superclass level with significant differences between the HG and PG groups at the superclass level. **(B)** Differential metabolite chord diagram. **(C)** Volcano plot of differential metabolites. **(D)** Heatmap analysis of correlation matrix plots based on gut microbiota metabolites for demonstrating the correlation between different compounds. Each circle in the graph represents a metabolite, and the color and size of the circle indicate the strength and magnitude of the correlation. **(E)** KEGG enrichment bubble plot. *X*-axis (Ratio): represents the enrichment ratio difference of a specific biological pathway between the two groups. A ratio greater than 1 indicates that the pathway is more enriched in PG. *Y*-axis (Terms): lists the names of various biological pathways involved in the analysis. Bubble size (Number): represents the number of significantly altered genes or proteins within each pathway. Larger bubbles indicate a greater number of significantly altered genes or proteins in that pathway. Bubble Color [–log_10_ (*p*-value)]: represents statistical significance using the negative logarithm of the *p*-value. Colors closer to red indicate higher statistical significance, meaning the pathway shows a more pronounced difference between the two groups. **(F)** Number of metabolites in the sample for different classes of lipids for HG vs. PG. **(G)** Top 20 enriched KEGG pathways in differential metabolites (VIP value ≥1, *p* < 0.05). The horizontal coordinates in the graph indicate the number of metabolites annotated under a particular KEGG pathway as a percentage of the number of all annotated metabolites, and the right side of the vertical coordinates is the KEGG Pathway primary classification, and the left side is the KEGG Pathway secondary classification.

The metabolite profiles of HG compared to PG in positive and negative ion modes were identified using LC-MS/MS to categorize the number of differential metabolites (Figure 4A). It was found that Lipids and lipid-like molecules (25.15%): this was the largest category of compounds in the samples in terms of percentage, including various lipids and lipid-like molecules such as fatty acids, triglycerides, etc. Organic acids and derivatives (24.17%): this was the second largest category, including various types of Organic acids and derivatives such as amino acids, nucleotides etc. Organoheterocyclic compounds (18.71%): this includes all organic cyclic compounds containing heteroatoms, such as indoles, purines, etc.

Chord plots based on the correlation coefficients of the differential metabolites can reflect the degree of correlation and association between the differential metabolites in the HG and PG samples. The top 20 differential metabolites were selected for the chord diagrams in descending order of *p*-value, the color of the dots represented different categories of metabolites, the size of the dots represented the size of log_2_ (Fold Change), the thickness of the connecting lines between metabolites represented the size of the correlation between the two metabolites, and the blue line represented negative correlation, and the red line represented positive correlation (only absolute values of the correlation coefficients were shown). The results are shown in Figure 4B. The metabolites that showed significant positive correlation between the different metabolites in the HG and PG samples with a strong degree of association were found to be Tecostanine (monoterpene alkaloid metabolite) and NEROL (monoterpene metabolite), Tecostanine and TRPM8 agonist WS-3, TRPM8 agonist WS-3 and TRPM8 agonist WS-3, and TRPM8 agonist WS-3 and TRPM8 agonist WS-3, and TRPM8 agonist WS-3 and NEROL agonist WS-3 with NEROL.

Volcano plot showing the expression of differential metabolites between the two groups of HG and PG. *X*-axis [log_2_ (Fold Change)]: indicates the logarithmic value of the fold change in metabolite expression levels. Positive values indicate up-regulation (UP) and negative values indicate down-regulation (DW). *Y*-axis [–log_10_ (*p*-value)]: indicates statistical significance, i.e., the logarithm of the negative logarithm of the *p*-value. Larger values indicate more significant results. Color of points: red points (UP: 456): indicate up-regulated metabolites, 456 in total. Blue dots (DW: 425): indicate down-regulated metabolites, 425 in total. Gray dots (NoDiff: 3,847): indicate metabolites with no significant change, 3,847 in total. Compared with PG, 456 up-regulated and 425 down-regulated metabolites were identified by HG (Figure 4C).

Heat map analysis of correlation matrix plots based on the correlation coefficients of the differential metabolites could reflect the correlation and the degree of association between the differential metabolites in the HG and PG samples, and the results were similar to chordal plots, which could show the correlation of the differential metabolite sites more comprehensively (Figure 4D). It was found that among the metabolites in the two groups, LysoPE was associated with 3,4-Dihydroxy-L-phenylalanine, Flibanserin was associated with L-Norleucine, STIRIPENTOL was associated with P-Toluenesulfonic acid, L-Norleucine was associated with Biotin-XX hydrazide and Caudoxiirene, Caudoxirene was significantly negatively correlated with P-Toluenesulfonic acid. The Riboflavin metabolism pathway was significantly up-regulated in HG and the Dopaminergic synapse pathway was significantly up-regulated in PG. Amino acid-related pathways: biosynthesis of amino acids and Phenylalanine, tyrosine, and tryptophan biosynthesis pathways were significantly up-regulated in HG. Pathways were significant in HG. Iron death-related pathway: the Ferroptosis pathway was also significant in HG. Retinol metabolism: the Retinol metabolism pathway was significant in PG.

Figure 4E presents the enrichment analysis results for various biological pathways in the study. As shown in the figure, pathways such as “Biosynthesis of amino acids” and “Protein digestion and absorption” exhibit higher enrichment ratios in PG and demonstrate greater statistical significance (indicated by redder colors). Conversely, pathways like “Cocaine addiction” show lower enrichment ratios and reduced statistical significance.

Figure 4F shows the results of the categorical annotation of metabolites using the LipidMaps database, which focuses on the distribution of metabolite counts by lipid class and its subclasses. HG has the highest number of metabolites in the fatty acyl group, the Fatty Acids and Conjugates [FA01] subclass, with 128 metabolites, compared to PG. In the Glycerophospholipids [GP] category, the subclass Glycerophosphocholines [GP01] had 18 metabolites, which was the more numerous subclass in this category. In the glycolipids (Sphingolipids [SP]), the subclass Ceramides [SP02] has 12 metabolites. In Sterols [ST], the subclass Sterols [ST01] has 27 metabolites, which is the more abundant subclass in this category.

Based on the differential metabolites in the two different groups, the KEGG classification of differential metabolites was plotted (Figure 4G). KEGG metabolic pathways can be classified into six categories, namely Cellular Processes, Environmental Information Processing, Genetic Information Processing, Metabolism, Metabolism, and Metabolism. Processing, Metabolism, and Organismal Systems.

## 4 Discussion

The present study investigated the changes in the composition and diversity of the intestinal flora and the metabolites of the intestinal flora before and after the entry of a specific group of young and middle-aged people into a high altitude area. In addition, the possible mechanisms of the changes in the intestinal flora of young and middle-aged people caused by high altitude were also investigated. The results showed that there were significant differences in the taxonomic composition and diversity of the intestinal flora of the young and middle-aged populations before and after entering the high-altitude area. The intestinal microbiota of young and middle-aged people at low altitude was more diverse, and the diversity of the intestinal flora was altered by the high altitude environment.

Our results showed that the composition of gut flora and differential flora were significantly different before and after entering high altitude in young and middle-aged populations. The gut microbiota composition of HG individuals showed the highest relative abundances at the phylum and genus levels for Firmicutes and *Blautia*, respectively. The results of differential flora at phylum and genus levels revealed that the relative abundance of Actinobacteriota and *Blautia* genera were significantly higher in HG than in PG. *Blautia* genera were significantly higher in relative abundance compared to PG. In plateau populations, *Prevotella* spp. are significantly enriched microorganisms in both Tibetan and Han Chinese populations, and when Han Chinese populations migrate from low to high altitude, their intestinal flora gradually converge to that of Tibetan populations, but when returning to low altitude, the intestinal flora reverses again (Han et al., 2024). Previous study (Zeng et al., 2020) comparing the intestinal microbial communities of Tibetans and Tibetan pigs living in high and low altitude environments found that the relative abundance of thick-walled phyla in the intestinal flora of the high altitude group was significantly higher. This change may be related to the increased energy demand of the host in the high-altitude environment and the regulatory role of microorganisms in host metabolism. In addition, the increase in the thick-walled bacterial phylum may also contribute to the maintenance of intestinal health and immune function of the host in a low-oxygen environment. An animal study found a significant increase in the relative abundance of thick-walled bacterial phylum in the gut flora of high-altitude ruminants (Wang X. et al., 2022). This change may be related to the challenge to host metabolism by the low oxygen and cold conditions in high altitude environments, and the increase in thick-walled bacterial phyla helps the host to obtain energy from food more efficiently and maintains the stability and functional redundancy of the intestinal microbial community. Specific genera within the thick-walled phylum (e.g., *Blautia* and *Faecalibacterium*) may play a key role in high-altitude acclimatization. A 108-day longitudinal study of 45 healthy Han Chinese adults found that the abundance of the genus *Blautia* A was significantly increased in high-altitude hypoxic environments (Su et al., 2024b). It suggests that the genus *Blautia* A plays an important role in the rapid response of the gut microbiome to the high-altitude hypoxic environment, and may maintain gut homeostasis through anti-inflammatory mechanisms and protection of the intestinal barrier, thus facilitating host adaptation to extreme environments. Our findings are consistent with previous studies, which suggest that the overall structure of the gut flora is mainly influenced by long-term dietary intake and extreme environments (Wang L. et al., 2024).

Our study also found that the α-diversity, differential metabolites and KEGG metabolic pathways of the intestinal flora of young and middle-aged people were more different after entering high altitude than before entering plateau. Animal studies found that the α-diversity of the intestinal flora of plateau pikas significantly decreased with increasing altitude, and the complexity of the colony co-occurrence network was reduced. KEGG analyses showed that colony metabolic pathways (e.g., energy metabolism, processing of genetic information) were significantly up-regulated, which supported the host's response to the plateau hypoxic environment. In addition, longitudinal studies in previous populations found (Su et al., 2024b) that the α-diversity of gut flora in high-altitude hypoxic environments declined rapidly at the beginning of the exposure, and the Shannon index remained below the baseline level at the end of the exposure. Despite an increase in low-abundance bacterial species during the later stages of acclimatization, the Shannon index of diversity of the whole microbial community did not return to the baseline level. When returning to lower altitudes, gut microbial diversity levels returned to baseline, suggesting that altitude change is strongly associated with gut microbial diversity. A study (Ma et al., 2025) that longitudinally tracked changes in the flora of 406 healthy men from low altitude (800 m) to high altitude (4,500 m) and analyzed the differences between acute (7 days) and long-term acclimatization (3 months) found that acute plateau exposure led to fluctuations in α-diversity (e.g., significant changes in the Shannon's index), with an increase in opportunistic pathogens (e.g., *Ruminococcus*), while beneficial bacteria (e.g., Faecalibacterium) decrease, and irreversible changes in β-diversity of the flora after prolonged exposure, with metabolic pathways (e.g., energy metabolism, amino acid synthesis) reconfigured to adapt to the hypoxic environment. Both our results and those of previous studies found significant effects of altitude change on both gut flora diversity and metabolic pathways. The high altitude environment can lead to a decrease in the α-diversity of the intestinal flora, and at the same time, the intestinal flora can help the host to adapt to the low oxygen environment by up-regulating energy metabolism and other related metabolic pathways. For different groups, Du et al. (2022) studied obese Tibetan children at different altitudes on the Tibetan Plateau and found that the α-diversity of the intestinal flora of children at high altitude (>3,600 m) was significantly lower than that of children at low altitude (<2,260 m). There are relatively few studies on the changes in gut flora at high altitude in young and middle-aged populations. It is possible that young and middle-aged people may have different adaptive capacities and changes in gut flora at high altitude compared to children and Tibetans. The molecular mechanisms of colony-host interactions (e.g., regulation of hypoxia signaling pathways by colony metabolites) still need to be explored in depth.

The diversity of the gut microbiota may be influenced by a number of factors. The gut microbiota is a complex ecosystem of trillions of microorganisms (bacteria and fungi) residing in the human gastrointestinal tract, which interact with host physiology. The phylum Thick-walled Bacteria and the phylum Anabaena dominate the gut flora of healthy hosts (Qiu et al., 2022), which break down carbohydrates and indigestible oligosaccharides in food and provide abundant energy to the intestinal epithelium. The gut microbiota is closely associated with human health and disease, and dysbiosis triggers a variety of chronic diseases such as infection, inflammation, cancer, digestive disorders, heart disease, lung disease, obesity, metabolic syndrome, type 2 diabetes mellitus, anxiety, depression, mood, regulatory behaviors, Alzheimer's disease, dementia, etc. (Zikou et al., 2024; Mousa et al., 2022), and is associated with sleep. Gut flora has a key role in defending against pathogens, regulating metabolic, endocrine and immune functions, and influencing drug metabolism and absorption (Silva et al., 2020). The brain is influenced by the gut microbiota through the gut-brain axis, a complex communication system connecting the gut to the brain, which acts through a combination of neural, immune and chemical signaling pathways, of which the vagus nerve is one of the important direct pathways. The immune system is also influenced by the gut and the brain, and the gut microbiota plays a key role in the physiology of the immune system, which produces chemicals such as short-chain fatty acids and neurotransmitters that directly influence brain function (Mhanna et al., 2024). Factors that influence the composition of the gut microbiota include diet, genetics, environment, geographic location, stress and antimicrobial agents (Cantón et al., 2024). The effect of high altitude on the gut flora is significantly altered in high altitude environments, with diversity and abundance being affected regardless of whether an individual is exposed for a prolonged period of time or an acute change in altitude. Similar changes were observed in the young and middle-aged population in this study 6 months after entering high altitude, further confirming the effect of high altitude on intestinal flora (Liu et al., 2024). With changes in altitude, the intestinal flora and host plasma metabolome undergo dynamic changes, which may be associated with the changes in intestinal flora observed in this study (Han et al., 2024). Possible mechanisms are explored for the effect of the hypoxic environment, which at high altitude may lead to the impairment of the intestinal mucosal barrier, affecting the survival environment of the intestinal flora, and thus affecting the composition and function of the intestinal flora (Liu et al., 2024). The hypoxic environment may also affect the immune function of the intestinal tract, which in turn affects the balance of the intestinal flora, and changes in the intestinal flora have been associated with memory dysfunction in high-altitude environments, suggesting that the hypoxic environment may affect the intestinal flora by affecting the intestinal immune function (Zhang et al., 2023). The changes in intestinal flora may be related to genetic background, which provides a reference for further research on the role of genetic factors in the changes of intestinal flora in young and middle-aged people after entering high altitude (Su et al., 2024b). Mood changes are easy to occur at high altitude, and some studies have shown that depressed mood can cause changes in the composition and diversity of intestinal flora (Hu et al., 2019). The significantly up-regulated pathway in PG in young and middle-aged populations suggests that dopaminergic synaptic activity may be more active at low altitude, which may be related to changes in energy metabolism and neural signaling. The significantly up-regulated pathway in HG suggested that riboflavin metabolism might be more active at high altitude, which might be related to the increased energy demand of cells. Amino acid synthesis may be increased in a high altitude environment, which may be related to cell growth and repair processes. Cells may be more susceptible to iron death in high altitude environments, which may be related to increased levels of oxidative stress. This is similar to previous findings, which found that purine metabolism is altered during high altitude acclimatization in humans, and that the levels of certain purine metabolites are altered in humans at high altitude compared to low altitude, suggesting that purine metabolism may play an important role in plateau acclimatization (Horn et al., 2019). There are also previous studies that found (Kumari et al., 2025) significant changes in amino acid metabolic pathways such as phenylalanine and tyrosine at high altitude, as well as up-regulation of glycerophospholipids and sphingolipids metabolism, suggesting that the cell membrane dynamically adjusts to cope with hypoxic stress. Another study found (Liu et al., 2023) that glycolysis was down-regulated in the plateau environment, while the lactate/amino acid-pyruvate-TCA pathway and fatty acid oxidation were up-regulated, supporting the hypothesis that fatty acid derivatives are metabolically active. We also found fatty acids and their derivatives (Fatty Acids and Conjugates [FA01]): the number of metabolites in this subclass was significantly higher in HG than in PG (128). In the high altitude environment, the human body responds to a series of metabolic changes in response to hypoxia, as well as changes in nutritional strategies, including the adjustment of energy metabolism (Stellingwerff et al., 2019). This suggests that the metabolism of fatty acids and their derivatives is more active in high altitude environments, which may be associated with increased energy metabolism requirements. Fatty acids are important components of biological membranes, and their active metabolism may reflect the dynamic adjustment of cell membranes to environmental stresses such as hypoxia. Previous studies on the adaptive remodeling of energy metabolism in skeletal muscle under acute severe hypoxia found that high-altitude hypoxia caused a significant decrease in adenosine levels in muscle, although the adenine nucleotide content was generally unchanged, and the purine nucleotide cycling activity was enhanced in order to maintain the ATP/ADP ratio, limiting energy consumption and acidification, and also explored the changes in fatty acid metabolism, pointing out that fatty acids may be one of the major sources of energy for skeletal muscle at high altitude in resting state (Qiu et al., 2022). One of the main energy sources for hypoxia in resting skeletal muscle at high altitude (Chicco et al., 2018), which is similar to our findings of active fatty acid metabolism in high altitude environment. Previous studies have found (Chicco et al., 2018) changes in the levels of substances related to fatty acid metabolism, such as acetylcarnitine, in muscle and increased oxidative phosphorylation respiration of muscle fibers stimulated by ADP in high altitude hypoxic environments, suggesting that fatty acid metabolism is more active in high altitude environments to satisfy the energy demand, which is similar to our findings that the increase in eicosanoids in the HG may be related to the inflammatory response of the body and the vasculature regulatory mechanisms, and that the overall metabolism of fatty acyls showed an up-regulation trend at high altitude. Glycerophospholipids are major components of cell membranes, and the increase in their metabolism may reflect the adjustment of the cell membrane structure to the stresses of the high-altitude environment. Qiu et al. (2025) found that plateau hypoxia affects lipid metabolism, pointing out that glycerophospholipid metabolism is related to the dynamic adjustment of cell membranes, and that changes in intestinal flora may affect plateau acclimatization through the phospholipid synthesis pathway. Previous studies by Liao et al. pointed out (Liao et al., 2016) that a variety of metabolites in human plasma were altered after high altitude exposure, such as increased levels of free fatty acids like linoleic acid and decreased levels of lysophosphatidylcholines (LysoPCs). Among them, LysoPCs mediate many cellular signaling pathways in monocytes/macrophages involved in inflammatory responses, which correlates with our findings of inflammatory responses and increased glycerophospholipid metabolism in the body at high altitude environments, among others. Sphingolipids play an important role in cell recognition, signaling and the differences in their metabolism may reflect different adaptive mechanisms in cell signaling between the two groups. Sterols such as cholesterol have important roles in cell membrane stability and signaling, and the higher number of PG metabolites may be related to the need for stability of cell membranes in low altitude environments. Ottolenghi et al. (2020) noted that hypoxic environments activate a variety of metabolic pathways, including sphingolipid metabolism. Sphingolipid metabolites such as sphingosine-1-phosphate (S1P) play a key role in hypoxic acclimatization by enhancing vascular barrier function, inhibiting apoptosis, and facilitating oxygen release. This fits with the importance of sphingolipids in cell signaling, suggesting that differences in sphingolipid metabolism at different altitudes reflect different adaptive mechanisms of cell signaling.

## 5 Conclusions

Through a longitudinal study on the changes of intestinal flora and metabolism in young and middle-aged people after entering high altitude for half a year, we found that there are significant differences in the taxonomic composition, diversity, metabolites and metabolic pathways of intestinal flora in this special group of young and middle-aged people at different altitudes and different time points. We can better understand the impact of high altitude environment on human health, and provide scientific basis for the health management of people in high altitude areas, and improve the changes of intestinal flora and digestive discomfort symptoms in plateau.

## 6 Strengths and limitations

The population of this study is young and middle-aged group, which is special; the time span of the study is relatively long; it explores the dynamics of the intestinal flora and the changing rules of its metabolism after the young and middle-aged group enters the high altitude area. The included population was only Northwest China, a relatively small geographical span and relatively small sample size. Insufficient stratification was done for high altitude elevation stratification. No comparison was made with the population after returning to the plains. Samples were not collected at different time points after reaching high altitude. In the future, longitudinal studies across time, ages and regions need to be carried out and combined with metabolomics and functional experiments to analyze dynamic changes in gut microbiota at different time points after high-altitude exposure. Multi-center and large-sample studies should be carried out, and the differences in gut flora changes in different populations at high altitude should be explored in depth. Combine with other histological techniques, such as transcriptomics, to comprehensively reveal the mechanisms of intestinal flora changes in high-altitude environments.

## Data Availability

The data presented in the study are deposited in the figshare repository, accession 10.6084/m9.figshare.30812828.
